# Testing the Cambridge Quality Checklists on a review of disrupted families and crime

**DOI:** 10.1002/cbm.1837

**Published:** 2012-11-29

**Authors:** Darrick Jolliffe, Joseph Murray, David Farrington, Claire Vannick

**Affiliations:** 1 Department of Criminology, Leicester UniversityLeicester, UK; 2 Department of Psychiatry, Cambridge UniversityCambridge, UK; 3 Institute of CriminologyCambridge, UK

## Abstract

**Background:**

Systematic reviews of the relationship between non-manipulated factors (e.g. low empathy) and offending are becoming more common, and it is important to consider the methodological quality of studies included in such reviews.

**Aims:**

To assess aspects of the reliability and validity of the Cambridge Quality Checklists, a set of three measures for examining the methodological quality of studies included in systematic reviews of risk factors for offending.

**Methods:**

All 60 studies in a systematic review of disrupted families and offending were coded on the CQC and codes compared with the effect sizes derived from the studies.

**Results:**

Overall, the CQC was easy to score, and the relevant information was available in most studies. The scales had high inter-rater reliability. Only 13 studies scored high on the Checklist of Correlates, 18 scored highly on the Checklist of Risk Factors and none scored highly on the Checklist of Causal Risk Factors. Generally, studies that were of lower quality had higher effect sizes.

**Conclusions:**

The CQC could be a useful method of assessing the methodological quality of studies of risk factors for offending but might benefit from additional conceptual work, changes to the wording of some scales and additional levels for scoring. Copyright © 2012 John Wiley & Sons, Ltd.

## Introduction

To date, systematic reviews of factors relating to crime have focused primarily on assessing the collective impact of intervention studies such as the impact of closed-circuit television (CCTV; Welsh and Farrington, 2007). However, systematic reviews – the rigorous summarising of evidence from a number of primary research studies – have also been used to evaluate the relationship between non-manipulated or naturally occurring factors and offending. Jolliffe and Farrington (2004), for example, conducted a systematic review of questionnaire-based measures of empathy and offending and found that low cognitive empathy was strongly related to offending, but low affective empathy was only weakly related to it.

Even though systematic review methodology reduces bias when compared with narrative reviews, it is essential to assess the quality of primary studies included in the review. For criminological intervention studies, this is usually performed using the Maryland Scientific Methods Scale (e.g. Farrington, 2003), but there are no agreed criteria by which to assess the methodological quality of studies of risk factors, or naturally occurring events (e.g. disrupted families; Deeks et al., 2003). A new set of devices, however, *The Cambridge Quality Checklists* (CQC) were designed by Murray et al. (2009) to help ‘identify high-quality studies of correlates, risk factors and causal risk factors for systematic reviews and meta-analyses’. The CQCs were developed using clear definitions of correlation (i.e. variables that have been shown to be associated with one another), risk factors (i.e. variables that predict the outcome because they have clear temporal ordering), and causal risk factors (i.e. risk factors that can change and, when changed, cause a change in the risk for the outcome; Kraemer et al., 2005).

### Scoring the Cambridge Quality Checklists

Table 1 shows the three CQCs, with the original *Correlate* and *Risk Factor Checklists* but with language changes made to the *Causal Risk Factor Checklis*t, to aid clarity. The main change to the wording of the *Causal Risk Factor Checklist* is to refer to ‘variation in the risk factor’ rather than ‘inclusion of a comparison group’, to highlight that the relevant risk factor variation might be dichotomous, categorical or continuous (and investigated in cross-sectional, case–control or prospective longitudinal studies).

**Table 1:** The Cambridge Quality Checklists

**Correlate score (out of 5)**	
Sampling	
1	Total population or random sampling
0	Convenience or case–control sampling
Response rates	
1	Response and retention rates ≥70% and differential attrition ≤10%
0	Response rate <70% or retention rate <70% or differential attrition >10%
Sample size	
1	Sample size ≥400
0	Sample size <400
Measure of correlate	
1	Reliability coefficient ≥.75 and reasonable face validity
	*or* criterion or convergent validity coefficient ≥.3
	*or* more than one instrument or information source used to assess correlate
0	None of the above
Measure of outcome	
1	Reliability coefficient ≥.75 and reasonable face validity
	*or* criterion or convergent validity coefficient ≥.3
	*or* more than one instrument or information source used to assess correlate
0	None of the above
**Risk factor score (out of 3)**	
1	Cross-sectional data
2	Retrospective data
3	Prospective data (or study of fixed risk factor)
**Causal risk factor score (out of 7)**	
1	Study without variation in the risk factor
	No analysis of change
2	Study with variation in the risk factor but inadequately balanced
	No analysis of change
3	Study without variation in the risk factor
	With analysis of change
4	Study with variation in the risk factor but inadequately balanced
	With analysis of change
5	Study with variation in the risk factor and adequately balanced
	No analysis of change
6	Study with variation in the risk factor and adequately balanced
	With analysis of change
7	Randomised experiment
	Targeting a risk factor

As Table 1 shows, the checklist for correlates has five items scored ‘1’ for study feature present or ‘0’ for study feature not present. These items draw reviewers' attention to how the sampling was undertaken, the response and retention rates of these samples, the overall sample size achieved and how the correlate and outcome were assessed.

The second checklist of the CQC is used to determine whether a variable is a risk factor. Risk factors are, by definition, correlates that precede the outcome, so this checklist draws reviewers' attention to the time-ordering of data in the study, with studies using cross-sectional data scored ‘1’, studies using time-ordered retrospective data ‘2’ and studies which use prospective longitudinal data, in which a risk factor is measured before the outcome scored ‘3’.

It is arguable that the third checklist of the CQC (for causal risk factors) is the most important aspect of the CQC as this was designed to assess the extent to which the risk factor is causally related to the outcome. The checklist draws attention to two key issues regarding assessment of causality in non-randomised studies. The first is the extent to which within-individual changes in the outcome (e.g. offending) are associated with within-individual changes in the risk factor (e.g. disrupted families). The second issue is the extent to which the study controls for alternative explanations of the findings. The CQC coding defines the highest quality studies (scored 7) as randomised experiments that target a specific risk factor. The highest quality non-randomised studies (scored 6) test whether variation in the risk factor is related to within-individual change in the outcome, while controlling for relevant confounding variables. Other studies are scored between 1 and 5. Reviewers using the CQC should explicitly list which confounders provide plausible alternative mechanisms for any observed relationship between the correlate and outcome. For greater detail about the CQC, scoring instructions and justification for the levels and scoring, see Murray et al. (2009).

### Our current study

The aims of our study were to test the performance of the CQC and based on its empirical application, suggest possible refinements for future use. For this, a meta-analytic review of the relationship between disrupted families and delinquency was used, given the large number of studies available on this topic, its theoretical importance to a number of criminological theories and the methodological issues that have been noted in previous studies (e.g., Wells and Rankin, 1991; Amato, 2001).

This is not the first review^1^ of the relationship between disrupted families and delinquency. Wells and Rankin (1991) conducted a meta-analysis of 44 effect sizes relating to disrupted families and delinquency. The overall correlation of *r* = 0.15 is approximately equivalent to a 15% difference in delinquency between those from disrupted or intact families. Additional reviews, with some variation in methods, have identified a similar level of effect (e.g. Amato and Keith, 1991; Amato, 2001). These reviews have also highlighted the substantial variation in effect size estimates attributable to study methods.

The objective of our systematic review was to examine the evidence on the effects of a ‘disrupted family’ on offending (e.g. official offence, self-, parent, teacher reported offending) in light of the scores on the CQC.

## Methods

### Inclusion/exclusion criteria

Studies were considered for inclusion if they investigated the impact on offending of a disrupted family (defined as permanent separation from either biological parent as a result of divorce, separation, death or any other reason^2^ but continuing to live with one biological parent). ‘Offending’ was defined as having committed chargeable offences, whether or not apprehended or charged. This offending outcome had to be quantitative, such that an effect size could be calculated. In addition, a minimum requirement of study design was that it should include comparison between individuals from a disrupted family (minimum *N* = 25) and a non-disrupted family (minimum *N* = 25).

### Adapting the Cambridge Quality Checklists for the current review

Murray et al. (2009) suggested that aspects of the CQC should be adapted by reviewers for the specific topic under investigation. Reviewers should detail, for example, the important covariates that studies should balance in order to be considered ‘adequately balanced’. Here, we considered studies ‘adequately balanced’ if they accounted for all three of the following: parental antisocial behaviour (e.g. parental criminality, drug/alcohol use), parental conflict and family income. These were considered important covariates because all three have been established as risk factors for offending (e.g. Farrington, 1995) but also associated with disrupted families (e.g. McCord, 1982). Therefore, these variables could plausibly account for any association identified between disrupted families and offending. A study was also considered adequately controlled if it controlled for two of the above important covariates and two in a set of other covariates. The latter were child IQ, child school achievement, child impulsiveness/hyperactivity, quality of parenting, supervision of child, educational attainment of either parent and/or social class of either parent, again all established risk factors for offending (e.g. Lipsey and Derzon, 1998) that could also increase the likelihood of disrupted families. In addition, for a study to be coded ‘adequately controlled’, all covariates had to be measured prior to the family disruption (Murray et al., 2009) to ensure the correct temporal ordering between potential confounders and family disruption.

### Search strategy

The search for relevant studies was based on (1) obtaining studies from the reviews of Wells and Rankin (1991), Amato and Keith (1991) and Amato (2001); (2) contact with leading researchers in the area; and (3) electronic database searches (details of the latter can be found in Table A1 online). This led to the identification of 108 potentially relevant studies, but effect sizes could only be calculated in 60 studies; these formed the sample for analysis.

## Results

### Coding the Cambridge Quality Checklists

Table 2 shows how the 60 studies included in the review were scored using the CQC. For example, 40 of these studies used total population or random sampling, so were scored ‘1’ on this item; 25 studies were assessed as having an adequate response rate on the checklist for correlates. Overall, six studies scored ‘0’ on the *Checklist for Correlates* and four scored the maximum of ‘5’. There were very strong inter-relationships (i.e. odds ratios of 11.5 to 39.0, *p* < .0001) between the individual item scores for sampling, response rates and sample size but less strong relationships between scores for generalisability and scores for the measures of correlates and outcomes (see online Table A2).

**Table 2:** Scoring of Cambridge Quality Checklists on studies of disrupted homes

**Items of correlate score**	Total (out of 60 studies)
Adequate sampling	40
Adequate response rates	25
Adequate sample size	49
Adequate measure of correlate	25
Adequate measure of outcome	5
**Total correlate score**	Total studies (*n* = 60)
Correlate score of ‘0’	6
Correlate score of ‘1’	11
Correlate score of ‘2’	13
Correlate score of ‘3’	17
Correlate score of ‘4’	9
Correlate score of ‘5’	4
**Risk factor score**	Total studies (*n* = 60)
Cross-sectional data	33
Retrospective data	9
Prospective data	18
**Causal risk factor score**	Total studies (*n* = 60)
Study without variation in the risk factor	
No analysis of change	
Study with variation in the risk factor but inadequately balanced	
No analysis of change	60
Study without variation in the risk factor	
With analysis of change	
Study with variation in the risk factor but inadequately balanced	
With analysis of change	
Study with variation in the risk factor and adequately balanced	
No analysis of change	
Study with variation in the risk factor and adequately balanced	
With analysis of change	
Randomised experiment	
Targeting a risk factor	

On the *Checklist for Causal Risk Factors*, all 60 studies scored ‘2’, indicating studies with variation in the risk factor but inadequate control, and no analysis of change; this was the lowest score possible in our review as we excluded studies without variation in the risk factor. Most studies were given the score of 2 because of inadequate control for relevant confounding variables or because there was no attempt to balance for relevant covariates. Only two studies (Skarohamar, 2009; MacArthur Violence Study, 2010) balanced for all three ‘important covariates’. Another reason that studies were not given higher scores on the checklist for causal risk factors was that, generally, covariates had not been measured before measurement of family disruption; not one study examined changes in offending both before and after family disruption.

### Relationships between checklists

There was evidence that studies using prospective data (with a Risk Factor Checklist score of ‘3’) scored higher on the *Checklist for Correlates* (*M* = 3.5, SD = 1.1) compared with studies using retrospective data (*M* = 2.1, SD = 1.3) or cross-sectional data (*M* = 1.9, SD = 1.2; *F* = 10.8, *p* < .05). It was not possible to undertake a similar analysis for the causal risk factor score because the score for this was 2 for all studies. Studies were, however, separated into those that applied no balancing variables (42), those that applied some balancing of variables (but none of the important covariates; 11 studies) and those that balanced for at least one important covariate (7).

The mean score on the *Checklist for Correlates* for studies that applied no balancing was 2.2 (SD = 1.4), for studies that balanced for some covariates (but none of the important covariates), it was 3.1 (SD = 1.3) and for those that balanced for at least one important covariate, it was 2.6 (SD = 1.0). The difference between those with no balancing variables and those with some was significant (*p* < .05), but the other differences were not.

### Inter-rater reliability

The inter-rater reliability of the items of the CQC was tested by having 43 (randomly selected) studies independently coded. The independent rater was provided only with the original CQC article (Murray et al., 2009) and the list of ‘important covariates’ for guidance. Table 3 shows the percentage agreement for the *Checklists for Correlates*. Overall, there was very high inter-rater agreement between the items. This was the highest for adequate sample size (only one disagreement) and the lowest (but still high) for good measure of the correlate.

**Table 3:** Inter-rater reliability of checklist for correlates

Correlate score	% Agreement	Chi squared	*p*
Adequate sampling method	90.7	28.5	0.0001
Adequate response rate	86.0	22.1	0.0001
Adequate sample size	97.7	37.6	0.0001
Good measure of correlate	83.7	18.9	0.0001
Good measure of outcome	90.7	7.8	0.005

There was 86% agreement on the *Risk Factor Checklist* score between the two coders across the 43 studies. Inter-rater reliability was strong (Kappa = .77 (*p* < .0001). Also, all 43 studies that were coded by the independent rater were scored ‘2’ on the *Causal Risk Factor Checklist*, which corresponded exactly with the original coding.

### The Cambridge Quality Checklists and the relationship between disrupted homes and offending

The relationship between disrupted homes and offending was used to examine additional aspects of the CQC, having calculated an effect size for each study that met the minimum inclusion criteria and undertaking meta-analysis. The overall effect size for all 60 studies was *d* = 0.26 (*z* = 10.8, *p* < .0001) in a random effects model, approximately equivalent to a 13% difference in offending between disrupted and intact homes. This is similar to the results of previous reviews by Wells and Rankin (1991) and Amato (2001).

For the 60 studies, the total score on the *Checklist for Correlates* was related to the total mean effect size at *r* = −.23 (*p* < .01). In addition, when the studies were dichotomized into those that scored high on the correlate score (scores of 3, 4 or 5) and those that scored low (score of 0, 1 or 2), the high scoring group had a significantly lower mean effect size (*d* = .20 compared with *d* = .34; Q between groups = 7.9, *p* < .005).

A series of analyses of variance conducted to examine the relationship between the individual items on the *Checklist for Correlates* and mean effect sizes confirmed this last finding. Forty studies classified as having adequate sampling, for example, had a significantly smaller effect size (*d* = .20), compared with 20 studies that did not have adequate sampling (*d* = .40) (Q between groups = 15.6, *p* < .0001). A significant difference was also evident when studies with an adequate response rate (*N* = 25; *d* = .20) were compared with those with inadequate sampling (*N* = 35, *d* = .31) (Q between groups = 4.8, *p* < .003). Studies that had an adequate sample size, a good measure of the correlate and a good measure of the outcome, did not have significantly lower mean effect sizes.

As expected, mean effect sizes also differed significantly (*p* < .0001) depending on the *Risk Factor Checklist* score (in an analysis of variance, Table 4). Prospective studies were found to have the lowest mean effect size, followed by studies that were cross-sectional. The largest mean effect size was for studies that employed retrospective data.

**Table 4:** Comparison of mean effect size to aspects of Cambridge Quality Checklists

Checklist of risk factors	*N*	ES	95% CI	Q between groups	*p*
Cross-sectional data	33	0.21	.19–.23	413.6	0.0001
Retrospective data	9	0.41	.39–.43		
Prospective data	18	0.18	.16–.19		
Level of Balance					
None	42	0.35	.30–.41	34.2	0.0001
Some but not important	7	0.06	−06–.18		
Important	11	0.08	−.02–.18		

CI = confidence interval, ES = effect size.

A similar pattern of results was identified when the *Checklist for Correlates* was dichotomised (scores of 4 and 5 being high) and combined with a dichotomous *Checklist for Risk Factors* (prospective v retrospective and cross-sectional). The small number of studies (*N* = 8) with high scores on both the *Checklist for Correlates* and the *Checklist for Risk Factors* had a smaller effect size (*d* = .25) than studies low on both (*n* = 37; *d* = .31). Studies that were mixed had the lowest effect sizes (high correlate, low risk factor *d* = .14 and low correlate, high risk factor = .17).

Unfortunately, because of the limited balance of covariates used by the studies in our review, it was not possible to compare the mean effect size according to scores on the *Checklist for Causal Risk Factors*. The last part of Table 4, however, shows the comparison between the mean effect size and a level of balance for each of the 60 studies. Studies that balanced for at least one (of the three) important covariates and studies that balanced for some, but not important covariates, had small and non-significant effect sizes; however, those with no balancing variables had moderate effect sizes. This difference was significant (Q between groups = 34.2, *p* < .0001).

## Discussion

Our study suggests that the CQC could be revised into a useful tool for assessing the quality of studies included in systematic reviews of risk factors for criminal behaviour. The three scales of the CQC were scored using information from the 60 studies included in a systematic review of the impact of disrupted homes and offending. Overall, the checklists were easy to score and, in most cases, the information that was needed to score the checklists was available in the original reports. The only exception to this general rule was information about differential attrition that was missing in a number of studies and led to their being downgraded on the ‘adequate response rate’ item on the *Checklist for Correlates*. Researchers could improve the quality of study reporting by adopting standardised epidemiological study guidelines (e.g. Elm et al., 2008) to overcome this problem. Consistent reporting of relevant study features would increase transparency and prevent studies from being downgraded on the CQC because of lack of information.

Only a small number of studies obtain high scores on the *Checklist for Correlates* and *Checklist for Risk Factors*, and not a single study met the criteria for being adequately controlled. Generally, this was either because studies did not balance for the most appropriate covariates or because covariates were not measured before family disruption, thus preventing attribution of causal order. Typically, however, studies that scored higher on the *Checklist for Correlates* also scored higher on the *Checklist for Risk Factors* and tended to balance for some covariates.

Further support for the CQC comes from the test of inter-rater reliability that was very high overall for the 43 studies.

The design of the original CQC was done *a priori* and with limited evidence available on appropriate cut-off points for scoring studies as high or low quality. There are currently three criteria for coding whether or not a study has a good measure of the correlate. Studies of disrupted homes most commonly met the criterion of ‘more than one instrument or information source used to assess correlate’. Multiple information sources might, however, be more important than use of multiple instruments, and finer grading on the checklist scores would be needed to capture this. Similarly, it might be better if a study with an achieved sample of 399 were not scored in the same way as a study with a much smaller sample (both *n* < 400). Future revisions of the CQC might benefit from moving from dichotomous to scaled scoring (i.e. 0, 1, 2, 3) on the *Checklist of Correlates*, to capture additional variability.

Some of these issues could, however, be resolved by reviewers providing topic-specific criteria for scoring certain items, as the original CQC scoring instructions suggest. Although it might be desirable to score multiple informants as a ‘good measure of the correlate’ for a review of disrupted families (where multiple viewpoints increase confidence), multiple measures might add more value if reviewing an individual characteristic such as empathy or impulsivity.

Given the limited sensitivity of the *Checklist for Causal Risk Factors* to studies with some balance for appropriate covariates, it might be worthwhile considering adding a level to this checklist. This checklist could be expanded to eight items to include a new score for ‘study with variation in the risk factor and partially balanced/no analysis of change’. This would allow for the upgrade of studies that balanced for some relevant covariates but fell short of being adequately controlled.

An additional approach to increase variability in causal risk factor scores might be for reviewers to specify a lower number of variables that must be balanced in order for a study to be classified as ‘adequately controlled’ on the CQC. In our current review, however, we thought that the variables that were identified as important (parental antisocial behaviour, parental conflict and family income) or potentially important provide a series of plausible alternative mechanisms whereby disrupted homes might be correlated but not causally related to offending. Table 5 shows a proposed revision of the *Causal Risk Factor Checklist*, incorporating the new language and a third level for studies that make some attempt to balance for some important covariates.

**Table 5:** Proposed Changes to Causal Risk Factor Checklist

	Causal risk factor score (out of 8)
1	Study without variation in the risk factor
	No analysis of change
2	Study with variation in the risk factor but inadequately balanced
	No analysis of change
3	Study with variation in the risk factor and partially balanced
	No analysis of change
4	Study without variation in the risk factor
	With analysis of change
5	Study with variation in the risk factor but inadequately balanced
	With analysis of change
6	Study with variation in the risk factor and adequately balanced
	No analysis of change
7	Study with variation in the risk factor and adequately balanced
	With analysis of change
8	Randomised experiment
	Targeting a risk factor

## Conclusion

The CQC has useful characteristics for collating essential methodological information from primary research studies included in meta-analytic reviews. Future research should explore empirical support for changing the scoring system for the *Checklist for Correlates* to a continuous measure and determine best cut-off points for the five items. The *Causal Risk Factor Checklist* might benefit either from additional levels or from reviewers specifying a lower number of variables needed to classify a study as controlled. Potential improvements to the CQC should not, however, draw attention away from the fact that there were few high-quality studies of the impact of disrupted families on offending. Future researchers should attempt prospective longitudinal studies (or use data available from existing longitudinal studies) to investigate in this field.

## Footnotes

1 Some consider it important to distinguish between a meta-analysis (i.e. where effect sizes are pooled but where studies may not have been identified systematically) and a systematic review (i.e. where studies are identified, located and appraised in a systematic manner and possibly subjected to a meta-analysis; e.g. Farrington and Petrosino, 2001). Using these definitions, the study of Wells and Rankin (1991) would be considered a meta-analysis and our current research a systematic review.

2 It should be noted that research on disrupted homes has been criticised for operationalising this as a dichotomous, structural variable (disrupted vs intact) as opposed to a functional variable (i.e. how well the family member or members function in meeting the needs of children; Van Voorhis et al., 1988; Juby and Farrington, 2001), but most research continues to treat this as a dichotomy.
