# Supplementary material for: Testing the Cambridge Quality Checklists on a review of disrupted families and crime
Source: Crim Behav Ment Health. 2012 Nov 29;22(5):303–14. doi: 10.1002/cbm.1837 (PMC3660786; doi:10.1002/cbm.1837)
Supplement: Supplementary file 1 [file cbm0022-0303-SD1.docx]

**Jolliffe, Murray, Farrington and Vannick**

**Testing the Cambridge Quality Checklists on a Review of Disrupted Families and Crime**

**^Additional material for online version^**

The electronic search strategy included 23 electronic databases (Table I appendix) and this resulted in the identification of 30 additional studies. Of the 108 studies 60 were included and 48 were excluded. Of the 48 excluded studies, 25 were excluded because not sufficient data was presented to allow for the calculation of an effect size (e.g. no standard deviation presented), 7 were excluded because it was not clear that the family was disrupted (e.g. poor family functioning), 6 were excluded because of overlap (e.g. same data presented in two studies), 4 were excluded because they were review articles, 3 were excluded because it was not clear that outcome was offending and 3 were excluded because the numbers were too low.

Table A1.

1. Articlefirst
2. Arts and Humanities Citation Index
3. Applied Social Science Index & Abstracts
4. Cambridge Journals Online
5. Conference Proceedings Citation Index
6. Criminal Justice Abstracts
7. Educational Resources Information Centre
8. Electronic Collections Online
9. Embase
10. Informaworld
11. IgentaConnect
12. ISI web of Knowledge
13. JSTOR
14. Oxford University Press e-Journals
15. PsychArticles
16. PsychExtra
17. Psychinfo
18. Sage Journals Online
19. Scopus
20. SpringerLink
21. SwetsWise
22. Wiley Online
23. ZETOC

Table A2 shows the odds ratios of the inter-relationships of the five items of the checklist for correlates. There were very strong relationships evident between the items which assessed the method of sampling, response rate and sample size, but less strong relationships when these measures of generalisability were compared to the measures of correlates and outcomes. An adequate response rate was positively related to a good measure of the outcome (OR=8.8*).However, adequate sampling was inversely related to a good measure of the outcome. A good measure of the correlate and a good measure of the outcome were related, but not significantly, likely because of the small number of studies which actually had a good measure of the outcome.

Table A2. Inter-relationships of items on checklist for correlates.

|  | Adequate | Adequate | Adequate | Good Measure | Good Measure |
| --- | --- | --- | --- | --- | --- |
|  | Sampling | Response | Sample Size | of Correlate | of Outcome |
| Sampling |  | 33.3*** | 39.0*** | 1.5 | 2.9 |
| Response |  |  | 11.5* | 1.6 | 8.8* |
| Sample Size |  |  |  | 0.8 | 1.3 |
| Correlate |  |  |  |  | 6.5 |
| Outcome |  |  |  |  |  |

*** = p<.0001, ** = p<.001, * = p<.05
